# The poverty impacts of improved cowpea varieties in Nigeria: A counterfactual analysis

**DOI:** 10.1016/j.worlddev.2019.05.027

**Published:** 2019-10

**Authors:** Julius Manda, Arega D. Alene, Adane H. Tufa, Tahirou Abdoulaye, Tesfamicheal Wossen, David Chikoye, Victor Manyong

**Affiliations:** a International Institute of Tropical Agriculture (IITA), PO Box 30258, Lilongwe, Malawi; b International Institute of Tropical Agriculture (IITA), PMB 5320, Oyo Road, Ibadan 200001, Nigeria; c International Institute of Tropical Agriculture (IITA), Nairobi, Kenya; d International Institute of Tropical Agriculture (IITA), Southern Africa Research and Administration Hub, PO Box 310142, Chelstone, Lusaka, Zambia; e International Institute of Tropical Agriculture (IITA), PO Box 34441, Dar es Salaam, Tanzania

**Keywords:** Endogenous switching regression, Counterfactual, Improved cowpea varieties, Nigeria, Poverty reduction

## Abstract

Adoption of improved agricultural technologies has long been recognized as critical for reducing poverty through increased productivity, incomes, and asset accumulation. Using a nationally representative survey data from a sample of over 1500 households in Nigeria, this paper evaluates the impacts of adoption of improved cowpea varieties on income and asset poverty reduction using an endogenous switching regression model. The results showed that adoption of improved cowpea varieties increased per capita household income and asset ownership by 17 and 24 percentage points, respectively. The results based on the observed and counterfactual income and asset distributions further showed that adoption reduced both income poverty and asset poverty by 5 percentage points. The paper concludes with a discussion of the policy options for increasing adoption and impacts of improved cowpea varieties in Nigeria.

## Highlights

• We examine the impact of improved cowpea adoption on poverty in Nigeria.
• We use a unique and recent nationally representative survey data.
• Adoption leads to an increase in household income and asset ownership.
• Poverty reduces with the adoption of improved cowpea varieties.

## 1 Introduction

Agricultural productivity growth has long been recognized as one of the most important and effective pathways through which agricultural research and technologies can increase rural incomes and reduce poverty (Gollin, Hansen, & Wingender, 2018). However, the link between agricultural research and poverty reduction is not straightforward as benefits may not be accrued uniformly across different income groups. In particular, the returns from agricultural productivity growth can be beneficial on average, albeit ineffective in improving the income of the most vulnerable and poor farmers, who are often constrained by structural barriers that make improved technologies inaccessible and less profitable for them (Wossen, Alene et al., 2019). Nevertheless, a plethora of empirical evidence suggests that agricultural productivity growth is extremely important for the development prospects of largely rural and agriculture-dependent countries in Sub-Saharan Africa (SSA). For example, the World Bank estimates show that GDP growth originating in agriculture is at least twice as effective in reducing poverty compared to the same magnitude of growth in other sectors of the economy (World Bank, 2007). Another study by Ligon and Sadoulet (2008) indicates that agricultural income growth has the largest impact on the poorest people in the poorest countries. In SSA, for example, Christiaensen, Demery, and Kuhl (2011) show that growth in the agricultural sector is at least three times more effective in reducing poverty compared to the same magnitude of growth in other sectors of the economy.

In this paper, we examine how agricultural research that leads to the development and dissemination of improved crop varieties can be a key driver of productivity growth and poverty reduction. Over the past 50 years, investments in crop genetic improvement by national and international agricultural research have led to the development and release of a number of productivity-enhancing improved crop varieties in many countries in SSA (Evenso 1960, Walker and Alwang, 2015). The adoption of such productivity-enhancing improved crop varieties is expected to reduce poverty directly by raising farm incomes and welfare of adopters through increased production for home consumption, higher gross revenues from sales, and lower production costs (Byerlee et al., 2011, De Janvry and Sadoulet, 2002, Moyo et al., 2007). Adoption of new and improved crop varieties can also reduce poverty indirectly through lower food prices and higher wages (Byerlee et al., 2009). A few recent studies show that adoption of improved agricultural technologies is important in reducing poverty in developing countries including in SSA (e.g. Ali and Abdulai, 2010, Alene et al., 2009, Becerril and Abdulai, 2010, Kassie et al., 2011, Kassie et al., 2018, Mendola, 2007, Renkow and Byerlee, 2010, Wossen, Abdoulaye et al., 2019).

In this study, we focus on the poverty reduction effects of adoption of improved cowpea varieties in Nigeria, the largest producer and consumer of cowpea in the world with an estimated 45% share of the global cowpea production and over 55% of the production in Africa (Alene, Abdoulaye, Rusike, Manyong, & Walker, 2015). Although the crop is largely produced by farm households as a staple food crop, it is fast becoming a major source of protein and cash income for these same households. The crop has between 22 and 30% protein, which makes it an important source of low-cost nutrition for the urban and rural poor who cannot afford meat and milk products. Given the importance attached to cowpea, increasing its productivity through adoption of improved varieties is therefore an essential policy objective in Nigeria. To this end, international and national research investments in Nigeria have developed and promoted improved cowpea varieties that are high yielding, drought tolerant, and resistant to striga, alectra and insect pests (Boukar et al., 2018, Singh et al., 2002). These efforts have resulted in the release of over 20 improved cowpea varieties in Nigeria since the early 1980s (NACGRAB, 2016).

Despite these major efforts and the importance of cowpea for rural livelihoods, there is a lack of comprehensive and rigorous evidence on adoption rates and impacts of improved cowpea varieties on poverty, a key evidence to justify investment in research on crop genetic improvement. In this regard, estimating the impacts of adoption of improved cowpea on household income and poverty is critical because it gives a measure of the extent to which the technology actually affects household welfare (de Janvry, Dustan, & Sadoulet, 2011).

Using a comprehensive household and plot level data, this paper estimates the poverty reduction effects of adoption of improved cowpea varieties in Nigeria. We aim to contribute to the literature in the following ways. First, unlike previous studies, we used asset ownership to construct an asset-based poverty measure (e.g. Awotide et al., 2015, Carter and Barrett, 2006). This is critical as asset-based poverty measures highlight the structural nature of poverty by focusing on the productive capacity of a household based on its resource stock (Liverpool-Tasie & Winter-Nelson, 2011). Second, most of the previous studies mentioned above mainly established causality between adoption and poverty reduction at the household level, but haven’t estimated aggregate poverty impacts in terms of the number of poor people lifted out of poverty. The few studies that have used this measure (e.g. Wossen, Alene et al., 2019, Zeng et al., 2015) focused on cassava and maize respectively and not on cowpea. Third, by estimating impacts on income directly, we overcome the reliance on often unreliable and sensitive demand and supply elasticities, which are required to translate household level productivity impacts to aggregate poverty impacts (e.g. Zeng et al., 2015). In doing so, our approach takes into account both direct and indirect mechanisms as our outcome indicator, income, captures both productivity and market price effects.

The rest of the paper is structured as follows. Section 2 deals with the survey design and data collection whereas Section 3 presents the empirical approach, definition of variables, and descriptive statistics. Section 4 presents and discusses the empirical results and the last section concludes with a discussion of the policy implications.

## 2 Survey design and data collection

The data for this study come from a nationally representative sample survey of 1525 cowpea producing households conducted in 2017. A survey questionnaire was designed using computer assisted personal interviewing (CAPI) based software called *Surveybe* and administered by trained enumerators who collected data from households through personal interviews. The survey was conducted in 10 states — Borno, Bauchi, Gombe, Jigawa, Kaduna, Kano, Katsina, Kebbi, Sokoto, and Zamfara — which represent about 75% of the total cowpea production in Nigeria. The above 10 states were grouped into two geopolitical zones: northeast and northwest These states mainly fall within the Sudan Savanna, which is the major agro-ecological zone for cowpea production in Nigeria. A multistage stratified sampling procedure was used to select the households. In the first stage, a list of villages and Local Government Areas (LGAs) used for conducting national census in Nigeria was obtained from the National Population Commission (NPC).

In the second stage, 25 and 13 LGAs were selected in each geopolitical zone using probability proportional to size (PPS) sampling (only 13 LGAs were selected in the northeast region because only three states were considered (Borno, Bauchi and Gombe) and this was due to the security problems experienced in that region during the survey). In the third stage, five cowpea producing villages were then randomly selected from each of the selected LGAs. A sampling frame was developed for cowpea-growing households in the selected villages with the help of the extension agents from the Agricultural Development Programme (ADPs). In the final stage, eight households were randomly selected from each selected village resulting in a total sample of 1525 households (995 households in the northwest region and 530 households in the northeast region).

The survey collected valuable information on several key socio-economic variables at both plot and household levels. Seed samples of the popular local and improved varieties were used to facilitate the interviews with farmers about whether and when they have adopted particular improved varieties. The improved cowpea varieties (ICV) considered in the study are presented in Table A1 in the appendix. To address measurement errors commonly encountered with self-reported plot sizes, we used Global Positioning System (GPS) devices to measure the area under cowpea varieties. Data were also collected on production systems, technology choices and preferences, input use, farmers’ patterns of resource use, and socioeconomic characteristics of the sample households.

## 3 Conceptual framework and empirical approach

We model the adoption of ICV under the assumption that farmers choose between ICV and local cowpea varieties. The decision to adopt ICV may however be endogenous as farmers usually self-select into adoption based on both observable and unobservable characteristics. Without controlling for this, the effects of adoption on the outcome variables (e.g. income and asset ownership) would be biased. To ensure that we account for endogeneity, we use the endogenous switching regression (ESR) model. The ESR model estimates two separate outcome equations for adopters and non-adopters along with a selection (adoption) equation simultaneously (Alene & Manyong, 2007).

Following Pitt (1983) and Fuglie and Bosch (1995), let the adoption of ICV be a discrete choice resulting from the maximization of a utility function. The expected utility arising from the adoption of improved cowpea varieties, $U_{A}$ is compared to the utility of non-adoption $U_{N}$. A farmer will adopt if $D_{i}^{*}=U_{A}>U_{N}$. $D_{i}^{*}$ is a latent variable that captures the expected benefits from the adoption choice with respect to not adopting and is determined by a set of exogenous variables, $Z_{i}$ and the error term $\mu_{i}$:

(1) $$D_{i}^{*}=Z_{i}\alpha +\mu_{i}\;where\;D_{i}=\left\{\begin{array}{c} 1\;\;\;if\;D_{i}^{*}>0\\ 0\;\;\;otherwise \end{array}\right)$$

If a farmer adopts improved cowpea varieties, $D_{i}$ = 1 and zero otherwise. Eq. (1) represents the selection or adoption equation.

The outcome equations, conditional on adoption, can be presented as two regimes following Alene and Manyong, 2007, Fuglie and Bosch, 1995, Di Falco et al., 2011 as:

(2a) $$Regime\;1\;\left(Adopters\right):y_{1i}=X_{1i}\beta_{1}+\varepsilon_{1i}\;if\;D_{i}=1$$

(2b) $$Regime\;1\;\left(Non-Adopters\right):y_{0i}=X_{0i}\beta_{0}+\varepsilon_{0i}\;if\;D_{i}=0$$

where $y_{1i}$ and $y_{0i}$ are the outcome variables for adopters and non-adopters respectively. The three error terms $\mu_{i}$,$\varepsilon_{1i}$ and $\varepsilon_{0i}$ are assumed to have a trivariate normal distribution with a mean vector zero and covariance matrix:

$$Cov\left(\varepsilon_{1i},\varepsilon_{0i},\mu_{i},\right)=\Sigma =\left[\begin{array}{ccc} \sigma_{1}^{2} & \sigma_{10} & \sigma_{1\mu }\\ \sigma_{10} & \sigma_{0}^{2} & \sigma_{0\mu }\\ \sigma_{1\mu } & \sigma_{0\mu } & \sigma_{\mu }^{2} \end{array}\right]$$

where $\sigma_{1}^{2}$ and $\sigma_{0}^{2}$ are the variances of the error terms in Eqs. (2a), (2b). $\sigma_{10}$ is the covariance of $\varepsilon_{1i}$ and $\varepsilon_{0i}$, $\sigma_{1\mu }$ represent the covariance of $\varepsilon_{1i}$ and $\mu_{i}$; and $\sigma_{0\mu }$ is the covariance of $\varepsilon_{0i}$ and $\mu_{i}$. It can be assumed that $\sigma_{\mu }^{2}$ is equal to 1 since α is estimable only up to a scaler factor (Maddalla, 1983). As $y_{1i}$ and $y_{0i}$ are not observed simultaneously, the covariance between $\varepsilon_{1i}$ and $\varepsilon_{0i}$ is not defined. This implies that the expected values of $\varepsilon_{1i}$ and $\varepsilon_{0i}$ conditional on sample selection is non-zero because the error term in the selection equation is correlated with the error terms in Eqs. (2a), (2b) and ordinary least squares estimates of coefficients $\beta_{1}$ and $\beta_{0}$ are biased. Sample selection occurs when factors not observed by the researcher but known to the farmer affects both technology choice and outcomes (Fuglie & Bosch, 1995). The expected values of $\varepsilon_{1i}$ and $\varepsilon_{0i}$ conditional on sample selection are non-zero and can be represented as:

(3a) $$E\left[\varepsilon_{1i}|D_{i}=1\right]=\sigma_{1\mu }\lambda_{1i}$$

(3b) $$E\left[\varepsilon_{0i}|D_{i}=0\right]=-\sigma_{0\mu }\lambda_{0i}$$

The inverse mills ratios or selectivity terms ($\lambda_{1i}$ and $\lambda_{0i}$) can be included in Eq. (2a), (2b) to correct for selection bias. We use the efficient full information maximum likelihood (FIML) estimation procedure to estimate the endogenous switching model described above. The FIML also generates correlation coefficients i.e. correlations of the error terms of the selection and outcome equations (corr (*ε*, *u*) = *ρ*). There is endogenous switching if $\rho_{A}$ or $\rho_{N}$ (which are correlation coefficients for adopters and non-adopters, respectively) are significantly different from zero (Abdulai & Huffman, 2014). The signs of the correlation terms have an important economic interpretation (Abdulai and Huffman, 2014, Fuglie and Bosch, 1995). If $\rho_{A}$ < 0, it implies positive selection bias, which suggests that farmers with above average income and assets, are more likely to adopt improved cowpea varieties. On the other hand, if $\rho_{N}$ > 0, it implies negative selection bias.

Although the model may be identified by construction through nonlinearities generated in the selection equation, it is important for the *Z* variables in the selection model to contain an instrument for a more robust identification. We use the average number of years the farmer has been aware of ICV as the selection instrument. It is envisaged that the farmers’ willingness to adopt would increase as they gradually become more cognizant of the benefits of ICV (Zeng et al., 2017). During the survey, farmers were asked which year they first knew or heard about a particular ICV variety. The number of years the farmer has known the ICV was then constructed as the difference between the year 2016 (the year before the survey was conducted) and the year a farmer first knew/heard about the ICV. It is important to admit that access to ICV seed is a necessary condition for a farmer to adopt because awareness alone may not essentially imply any knowledge of the characteristics of the technology (Diagne and Demont, 2007, Dontsop Nguezet et al., 2013). Notwithstanding, some studies (e.g. Lunduka et al., 2012, Negatu, 2002) have shown that improved variety knowledge is important for adoption. We believe that the years that a farmer has been aware is a good proxy for the knowledge of the characteristics of various ICVs being promoted in northern Nigeria. We establish the admissibility of the instrument by performing a simple falsification test: if a variable is a valid selection instrument, it will affect the decision to adopt ICV, but will not affect the outcome variables among non-adopting farm households (Di Falco et al., 2011). Table A2 in the appendix shows that the average number of years the farmer has been aware of ICV can be considered a valid instrument: it is statistically significant in the selection equation but not significant in the income and asset ownership equations. Further, since our instrument actually exploits exogenous variation in time lag (i.e., from the point of awareness to adoption), it would arguably be exogenous to current levels of productivity and income. To underscore the relevance of our instrument, we have included a test on the relevance of our instrument (first stage regression) in Table 3. The results show that the selected instrument is relevant as it is significant at 1% significance level.

To estimate the impact of adoption of ICV on household incomes and asset ownership, we first specify the expected values of the outcome variables. For an adopter of ICV, the expected value of the outcome variable is expressed as:

(4) $$E\left(y_{1i}|D_{i}=1\right)=X_{1i}\beta_{1}+\sigma_{1\mu }\lambda_{1i}$$

The expected values for the same farmer had he/she decided not to adopt ICV (counterfactual) is given as

(5) $$E\left(y_{0i}|D_{i}=1\right)=X_{1i}\beta_{0}+\sigma_{0\mu }\lambda_{1i}$$

The impact of adoption on the outcome variables for those who adopted ICV—i.e. the average treatment effect on the treated (ATT)—is calculated as the difference between Eqs. (4), (5)

(6) $$\mathrm{ATT}=E\left(y_{1i}|D_{i}=1\right)-E\left(y_{0i}|D_{i}=1\right)=X_{1i}\left(\beta_{1}-\beta_{0}\right)+\left(\sigma_{1\mu }-\sigma_{0\mu }\right)\lambda_{1i}$$

### 3.1 Measurement of impacts on poverty and asset poverty

We used the ESR model to estimate the observed and counterfactual income distributions (Eqs. (4), (5)) which were then exploited to measure the impact of adopting ICV on poverty and asset poverty. To estimate poverty in our sample, we used the Foster, Greer, and Thorbecke (1984) indices^1^ defined as:

(7) $$P_{\alpha }=\frac{1}{N}\sum_{i=1}^{q}{\left[\frac{z-y_{i}}{z}\right]}^{\alpha }$$

where *N* is the total number of households, *q* is the number of poor households, $y_{i}$ is the household welfare measure (i.e. income per capita/day in our case adjusted for inflation), *z* is the poverty line and α is a parameter of inequality aversion. It follows that when α = 0 the formula reduces to the headcount index which shows the proportion of the population that lives below the poverty line. When α = 1, $P_{\alpha }$ is the poverty gap index, which measures the average poverty gap in the population as a proportion of the poverty line (where the non-poor have zero gaps); and when α = 2, $P_{\alpha }$ measures the severity of poverty and reflects the degree of inequality among the poor. The FGT class of poverty measures satisfies a convenient decomposability property (Ray, 1998). In our case, the FGT indices are appropriate because they allow us to assess poverty on the observed and counterfactual income scenarios. We use two poverty lines to estimate poverty and assess the robustness of our method (US$1.9,^2^ and US$ 3.5). The first one is the revised current international poverty line of US$1.9 per day at 2011 purchasing power parity conversion factors (PPPs) from the previous US$1.25 per day at 2005 PPPs (Ferreira et al., 2016). The second one is the lower middle income class poverty line at 2011 PPPs. According to the World Bank (2018), Nigeria is ranked as a lower middle income country based on the gross national income (GNI) per capita.

Asset poverty can be viewed as a household’s failure to have access to adequate wealth resources to meet basic needs for a certain period of time (Awotide et al., 2015). To measure asset poverty, we used the relative poverty line since there is no established asset poverty line in Nigeria. We calculated the relative poverty line as two thirds of the mean value of assets (US$234) owned by the sample households (Awotide et al., 2015). We also used the mean value of assets (US$350) to assess the robustness of our results. Eq. (7) was then used to calculate the asset headcount, asset gap and asset severity indices.

### 3.2 Descriptive statistics

We draw on the vast literature on adoption and impacts of improved agricultural technologies to identify explanatory variables (Ali and Abdulai, 2010, Becerril and Abdulai, 2010, Feder and Umali, 1993, Feder et al., 1985, Kassie et al., 2014, Khonje et al., 2015, Mason and Smale, 2013, Zeng et al., 2015). We present the definition and descriptive statistics for the selected variables in Table 1. Variables that capture household welfare include yield, household income and asset ownership characteristics. For productivity enhancing technologies such as ICV, adopters are expected to realize more yields which consequently should result in increased household income and asset ownership. The average household income — which includes cash income from crops, livestock and livestock products, and off-farm income (salaries, remittances, farm labour wage income, pension income and income from business) — was approximately $662 per capita/year with an average per capita income of US$1.811 per day.

**Table 1:** Variable names, definitions, and descriptive statistics.

Variable	Definition	Mean	SD
Yield (kg/ha)	Average cowpea production per hectare	643.204	427.040
Household income	Total real household income per capita per year (US$)	661.183	537.722
Household income	Total real household income per capita per day (US$)	1.811	1.473
Asset ownership	Value of household assets per capital (US$)	350.678	479.570
Adoption of improved cowpea varieties	=1 if household planted improved cowpea varieties in the 2016 cropping season, 0 otherwise	0.415	0.493
Age of the household head	Age of the household head in years	44.10	12.12
Sex of the household head	=1 if household head is male, 0 otherwise	0.961	0.193
Education	=1 if attended junior secondary school, 0 otherwise	0.035	0.183
Adult males	Number of male adults in prime age group (15–59 years)	2.284	1.537
Adult females	Number of female adults in prime age group (15–59 years)	1.843	1.057
Total cultivated land	Total land cultivated by household in hectares	4.729	5.144
Access to off farm income	=1 if household has access to off farm income, 0 otherwise	0.849	0.358
Crop marketing	=1 if member of crop marketing group, 0 otherwise	0.007	0.080
Implement index	Agricultural implement index	−0.007	1.261
Information index	Agricultural information index	0.002	1.402
Number of donkeys	Number of donkeys owned by household	0.055	0.413
Credit constrained	=1 if farmer is credit constrained, 0 otherwise	0.317	0.466
Time to output market	Time in minutes to output market	40.92	63.37
Distance to seed dealer	Distance to seed market in minutes of walking time	69.14	143.7
Years aware	Number of years the farmer has been aware of the ICV	2.946	3.644

Household total productive assets include household assets (e.g. furniture, radios and TVs), productive assets (e.g. farm implements, oxcarts, ploughs and sprayers), and livestock assets (e.g. cattle, pigs, goats, sheep, and donkeys) similar to the assets considered by Liverpool-Tasie and Winter-Nelson (2011). On average, sample households had assets with a value of US$350.

About 42% of the households planted ICV in the 2016 cropping season. Household characteristics were captured by variables such as age, sex, education, cultivated land, number of adult females and males in the household and access to off-farm income. About 96% of the households were male-headed, with about 4% of the households attending junior secondary school education. Land is an indicator of resource endowment for the household and on average farmers cultivated 4.73 ha.

The number of adult females and males between the ages of 15 and 59 are proxies for household labor endowment. Almost 85% of the sample households had access to off-farm income. This may affect the individual household’s labour allocation and cash earnings and is also an indication of the dependence on off-farm employment in the household’s community and among neighboring communities (Smale & Mason, 2014). We proxy ownership of agricultural implements and access to information using agricultural implement and information indices constructed using principal component analysis (PCA). The agricultural implements that were considered include ploughs, hoes, and ox/donkey carts among others. In the construction of the information indices, we considered all the information sources related to improved varieties and agronomic practices. Sources of information included farmer/cooperative groups, extension agents, neighbors/relatives, research centers and radio/TV. We conducted PCA on the agricultural implement holdings and information sources to reduce the dimensionality into single scores for implements and sources of information. Specifically, we used the first principal component because it explains the most variance in the data as opposed to multiple components. The factor scores from the first component were used as weights for each implement/information source in order to construct the indices for each individual household.

Membership in crop marketing groups, distances to the output markets and seed dealers are important indicators of market characteristics. It takes an average of 41 minutes for farmers to transport produce to the market and about 69 minutes to access the market for inputs such as seed. Lastly, Table 1 indicates that on average most of the households have been aware of ICV for close to three years.

Table 2 displays the means of variables by adoption status (1 = adopters and 0 = non-adopters). The results in Table 2 show that adopters obtained more yields per hectare, compared with non-adopters, although the difference is not significant. Adopters of ICV had on average higher incomes per day (US$ 1.918) compared with the non-adopters (US$1.736). Results also show that adopters were significantly distinguishable in terms of household characteristics such as sex and education of the household head. About 97% of the adopters and 95% for non-adopters were headed by males while about 2.4% more adopters than non-adopters attended junior secondary school education. The number of years that adopters were aware of ICV (5.13) was more than that for non-adopters (1.396).

**Table 2:** Farm and household characteristics by adoption status.

Variable	All	Adopters (N = 633)	Non-adopters (N = 892)	Difference
Yield (kg/ha)	643.204	659.450	633.440	26.010
Household income (US$/year)	661.183	700.129	633.546	66.582**
Household income (US$/day)	1.811	1.918	1.736	0.182**
Asset ownership (US$/year)	350.678	374.592	333.708	40.884
Age of the household head	44.10	44.084	44.113	0.030
Sex of the household head	0.961	0.973	0.952	0.020**
Education	0.035	0.049	0.025	0.024**
Adult males	2.284	2.322	2.257	0.066
Adult females	1.843	1.880	1.816	0.064
Total cultivated land	4.729	4.746	4.717	0.030
Access to off farm income	0.849	0.863	0.840	0.023
Crop marketing	0.007	0.006	0.007	−0.000
Implement index	−0.007	0.085	−0.073	0.158**
Information index	0.002	0.072	−0.048	0.120
Number of donkeys	0.055	0.074	0.043	0.032
Credit constrained	0.317	0.305	0.326	0.021
Time to output market	40.92	39.628	41.814	2.186
Distance to seed dealer	69.14	73.567	66	−7.567
Years aware	2.946	5.130	1.396	3.734***

The difference is measured by the two-sample *t-*test with equal variances.

^*^p < 0.10.

** p < 0.05.

*** p < 0.001.

**Table 3:** Distributional summary statistics for income and asset ownership.

	Real per capita income (US$/year)				Asset ownership (US$/year)			
Quantile group	Adopters		Non-adopters		Adopters		Non-adopters	
	Quantile	Share, %	Quantile	Share, %	Quantile	Share %	Quantile	Share, %
1	184.099	1.959	170.97	1.867	44.432	0.65	44.68	0.758
2	262.545	3.279	244.34	3.335	81.243	1.646	79.001	1.851
3	347.874	4.312	299.115	4.319	123.988	2.688	111.6	2.882
4	434.227	5.654	380.899	5.334	173.152	4.001	151.249	3.918
5	521.614	6.782	495.604	6.851	230.272	5.313	195.942	5.167
6	636.22	8.27	603.973	8.74	297.58	7.001	267.014	6.97
7	799.052	10.388	735.696	10.544	363.532	8.943	345.313	9.06
8	1050.203	12.972	952.858	12.989	544.809	11.822	463.464	12.18
9	1443.77	17.317	1304.597	17.484	790.897	17.733	699.696	16.854
10		29.067		28.537		40.204		40.359

Table 3 presents the distribution of household income and asset ownership by the adoption of ICV. The population of the sample households was split into tenths ordered by income and asset ownership (decile groups) and the estimates shown are for the nine deciles (p10; p20; p30; p90). It is clear from Table 3 that adopters had more income and assets in all the decile groups as compared to non-adopters. The results in Table 3 further show that the poorest tenth of the sample households received about 2% (adopters) and 1.9% (non-adopters) of the total income as compared to the richest tenth who received 29% of the total income. So, the poorest adopters received slightly more income than the non-adopters. The distribution of the assets can also be interpreted in a similar way.

## 4 Empirical results and discussion

### 4.1 Determinants of improved cowpea adoption and impact on household income and asset ownership

Table 4 presents the full information maximum likelihood estimates of the ESR model. Results from the selection equation indicate that age, education, access to information and the number of years a farmer has been exposed to ICV are important determinants of adoption of ICV. The age of the household head and its square were significant determinants of adoption, implying that age has a non-linear effect on adoption of ICV. The results also show that farmers who completed at least 6 years of education were more likely to adopt ICV. Education has been widely cited as an important determinant of adoption of improved agricultural technologies in Africa with the main reason behind its importance being that educated farmers have better access to information and are able to understand the importance and benefits of growing improved varieties (Abdulai, 2016, Alene and Manyong, 2007, Foster and Rosenzweig, 2010, Manda et al., 2018). Information plays a very important role in the adoption of improved agricultural technologies as it is expected that farmers will only adopt an improved variety if they have enough information about the benefits of a particular technology (Adegbola & Gardebroek, 2007). The adoption of ICV is lower in the northeast region and this may reflect the unobservable differences in terms of the resources and weather pattern. This may also reflect the security problems being faced in the north eastern part of Nigeria which has impacted negatively on agricultural production in the area.

**Table 4:** Full information maximum likelihood estimates of the endogenous switching regression model.

Variable		Household income		Asset ownership	
	Selection	Non-adopters	Adopters	Non-adopters	Adopters
Age of the household head	−0.032* (0.018)	−0.050*** (0.012)	−0.016 (0.015)	−0.01 (0.02)	−0.02 (0.02)
Age of the household head squared	0.000* (0.000)	0.000*** (0.000)	0.000 (0.000)	0.00 (0.00)	0.00 (0.00)
Sex of the household head	0.278 (0.200)	0.081 (0.113)	0.146 (0.175)	0.22 (0.16)	0.42 (0.26)
Education	0.449** (0.197)	−0.090 (0.153)	−0.072 (0.132)	−0.47** (0.22)	−0.43** (0.19)
Number of male adults	−0.017 (0.027)	−0.111*** (0.018)	−0.105*** (0.021)	−0.08** (0.03)	−0.10** (0.03)
Number of female adults	0.023 (0.037)	−0.116*** (0.024)	−0.016 (0.030)	−0.08** (0.03)	−0.01 (0.04)
Ln cultivated land	−0.031 (0.054)	0.066* (0.035)	0.061 (0.045)	0.30*** (0.05)	0.18** (0.07)
Access to off farm income	0.108 (0.105)	0.470*** (0.066)	0.440*** (0.082)	−0.09 (0.09)	0.04 (0.12)
Crop marketing	0.121 (0.431)	0.041 (0.288)	0.759** (0.350)	0.01 (0.41)	0.68 (0.52)
Implement index	0.028 (0.030)	0.072*** (0.020)	0.097*** (0.024)	0.34*** (0.03)	0.40*** (0.04)
Information index	0.045* (0.026)	0.037** (0.018)	−0.015 (0.019)	0.01 (0.03)	−0.04 (0.03)
Number of donkeys	0.058 (0.088)	−0.026 (0.056)	−0.138* (0.071)	0.10 (0.08)	0.30** (0.11)
Credit constrained	−0.012 (0.078)	−0.124** (0.051)	−0.188** (0.061)	−0.18** (0.07)	−0.27** (0.09)
Time to output market	−0.000 (0.001)	−0.000 (0.000)	−0.001* (0.000)	0.00 (0.00)	0.00 (0.00)
Ln distance to seed market	0.010 (0.034)	−0.015 (0.024)	−0.058** (0.026)	0.05 (0.03)	−0.04 (0.04)
Years aware	0.211*** (0.011)				
Northeast	−0.166** (0.079)	0.106** (0.050)	−0.074 (0.064)	0.16** (0.07)	0.06 (0.09)
Constant	−0.462 (0.475)	1.427*** (0.307)	1.058** (0.386)	4.98*** (0.44)	5.72*** (0.57)
*Model diagnosis*					
*ρ*0		−0.071 (0.086)		−0.11 (0.09)	
*ρ*1			−0.225** (0.114)		−0.23** (0.12)
Likelihood ratio test of independent equations *χ2*(2)		4.64*		5.52*	
Observations	1525	633	892	633	892

Standard errors in parentheses.

* *p* < 0.10.

** *p* < 0.05.

*** *p* < 0.001.

The aim of the selection equation is not to perfectly explain adoption, but to account for unobserved heterogeneity that could bias the impacts derived from the outcome equations (Kabunga, Dubois, & Qaim, 2012). To account for any unobserved heterogeneity, we included an instrument (the average number of years the farmer has been aware of ICV) in the selection equation and not in the outcome equations.

The likelihood ratio tests for the joint independence of the three equations and correlation coefficients are also displayed in Table 4. The test results show that the equations are dependent, hence if we had assumed that these equations were independent, biased estimates would have been obtained. As mentioned earlier, the correlation coefficients have an important economic interpretation. In both the income and asset equations, only the coefficient for adopters (*ρ*1) was significant, and this implies that there was endogenous switching, therefore ICV adoption may not have the same effect on the non-adopters, if they choose to adopt. The negative sign on *ρ*1 suggests positive selection bias implying that farmers with above-average incomes and assets have a higher propensity of adopting ICV. This is highly consistent with earlier studies (e.g. Abdulai and Huffman, 2014, Alene and Manyong, 2007, Manda et al., 2017). Results^3^ for the outcome equations are shown in columns 3 and 4 for income and 5 and 6 for asset ownership.

Table 5 presents the estimated ATTs (impact) of adoption of ICV on household income and asset ownership from Eq. (6). The results show that the causal effect of adopting ICV was about US$0.22 per capita/day, which is equivalent to a 17 percentage-point increase in household income. This implies that current adopters would have foregone almost US$80 (US$0.22/day*365 days) per year per capita had they not adopted ICV. Similarly, adoption of ICV led to an average 24 percentage-point increase (US$50) in the value of household assets per capita. These results are consistent with the findings of Abdulai, 2016, Awotide et al., 2015, Zeng et al., 2015 in Zambia, Nigeria and Ethiopia, respectively.

**Table 5:** Treatment effects: Endogenous switching regression model.

Outcome variables	Decision stage		Treatment effect
	To adopt	Not to adopt	ATT
Household income (US$/capita/day)	1.526	1.308	0.217*** (0.029)
Asset ownership (US$/capita/year)	254.218	204.306	49.911*** (9.791)

Standard errors in parentheses.

*** *p* < 0.001.

### 4.2 Impact on income poverty and asset poverty in Nigeria

**Fig. 1:**
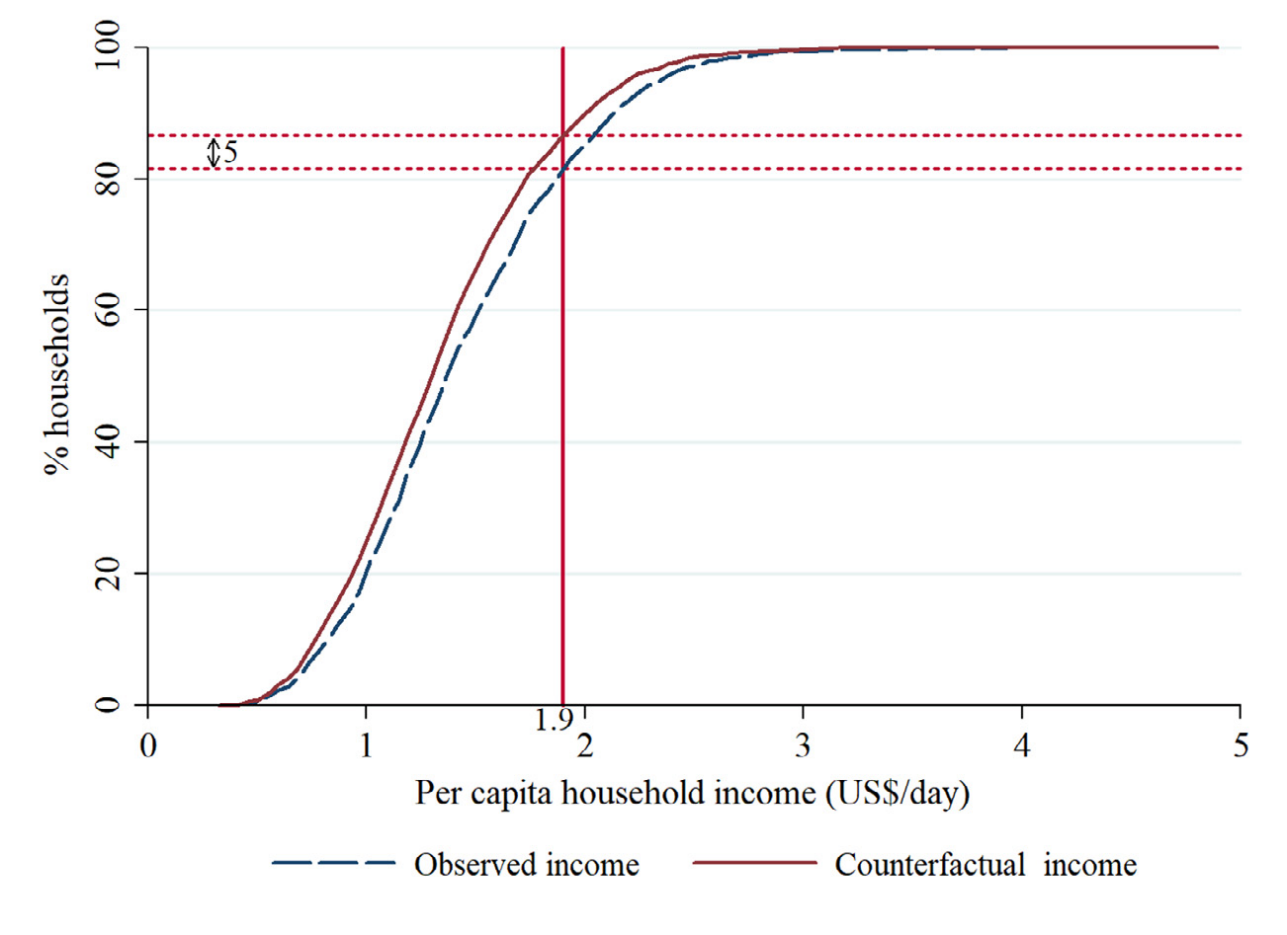
Observed and counterfactual income distribution for sample households.

**Fig. A1:**
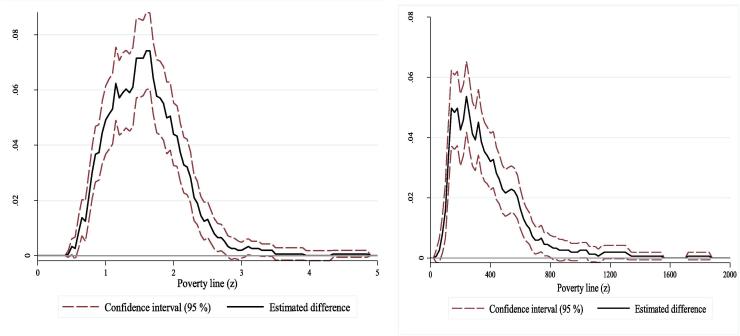
Observed and counterfactual income and asset ownership distribution differences.

To estimate the effect of adoption on poverty reduction, it is necessary to know the outcome for the adopting farmers if they had not adopted. We therefore used the ESR model to estimate the observed and counterfactual income distributions (Eqs. (4), (5)). The approach based on the observed and counterfactual income distributions to measure the impact of adoption on poverty is similar to the methodology used by Zeng et al., 2015, Larochelle et al., 2015. Fig. 1 shows the FGT (α = 0) cumulative distribution functions (CDFs) for the observed and counterfactual household per capita daily incomes for the sample households. The graph indicates that the observed income distribution first order stochastically dominates the counterfactual income distribution. Fig. A1 in the appendix plots the differences between these two graphs and it shows that for most parts of the graph, the difference is non-zero. Applying the international poverty line of US$1.9 per person per day, the results show that 87% of the households would have been poor had they not adopted ICV while only 82% were poor with adoption. This implies that adoption of ICV reduced poverty by 5 percentage points among the sample households.

In addition to the information provided in Fig. 1, Table 6 shows a similar pattern with regards to the depth and severity poverty indices, with the observed income distribution showing lower poverty indices as compared to the counterfactual distributions. The poor have on average an income shortfall of 31% of the poverty line in the counterfactual scenario compared to 28% with adoption of ICV (depth of poverty). Similarly, assuming equal transfers to the poor, the cost of eliminating poverty per year would be higher without adoption (US$215^4^) than with adoption (US$194). The results are quite robust at the other poverty line of US$3.2, which indicate that adoption of ICV reduces poverty by 0.3 percentage points (Table 6). The results show a similar pattern with regards to the depth and severity poverty indices, with the observed income distributions showing lower poverty indices as compared the counterfactual scenarios. Finally, column 6 of Table 6 shows the percentage of people escaping poverty due to the adoption of ICV. The results show that about 6% of the poor cowpea producers escaped poverty in the 2016 production season due to adoption of ICV.

**Table 6:** Poverty impacts of improved cowpea varieties on poverty reduction.

Poverty line (US$ per person per day)	FGT index	Observed	Counterfactual	Poverty impact	Percent of poor escaping poverty^1^
1.9	Headcount	0.816	0.866	0.050	5.8
	Depth	0.276	0.311	0.035	
	Severity	0.120	0.141	0.021	
3.2	Headcount	0.996	0.999	0.003	0.3
	Depth	0.551	0.579	0.028	
	Severity	0.329	0.357	0.028	

1 Note: This is calculated by dividing the poverty impact by the counterfactual headcount index.

**Fig. 2:**
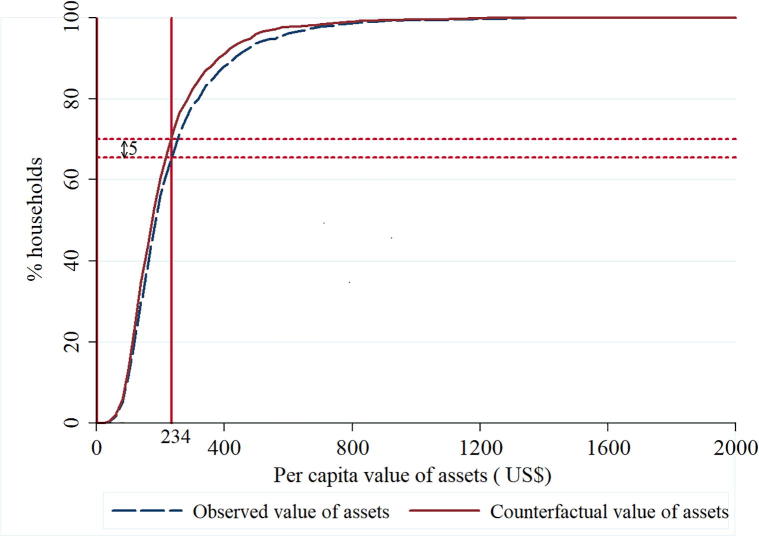
Observed and counterfactual asset distributions for sample households.

Similar to the poverty results above, Fig. 2 shows the observed and counterfactual per capita asset distributions. The relative asset poverty lines were calculated as two-thirds of the mean value of the assets (US$234) and the mean value of the assets (US$305). The results show that reduction in asset poverty ranged from 4 to 5%, with highest reduction observed at the relative poverty line of US$234. Both the observed (66%) and the counterfactual (71%) asset poverty rates were lower than poverty headcounts above. So even though the percentage-point poverty reduction was the same in both cases, the asset poverty rates were relatively lower than the poverty rates based on income.

Results in Table 7 further indicate that the depth and severity of asset poverty reduced by about 2.6 and 1.5 percentage points, and almost 7% of the asset poor households escaped asset poverty at the US$234 asset poverty line.

**Table 7:** Impacts of improved cowpea varieties on asset poverty.

Asset poverty line (US$ per year)	FGT index	Observed	Counterfactual	Poverty impact	Percent of poor escaping asset poverty^1^
234	Headcount	0.655	0.707	0.052	7.4
	Depth	0.245	0.271	0.026	
	Severity	0.117	0.132	0.015	
350	Headcount	0.839	0.876	0.037	4.2
	Depth	0.417	0.449	0.032	
	Severity	0.242	0.266	0.024	

1 Note: This is calculated by dividing the poverty impact by the counterfactual headcount index.

Applying a procedure similar to the one used by Alwang and Siegel, 2003, Zeng et al., 2015, Wossen, Alene et al., 2019, Wossen et al., 2017, Manda et al., 2017, the percentage point reduction in the income poverty and asset poverty headcount indices estimated in Fig. 1, Fig. 2 can be used to estimate the number of people who have been lifted out of poverty due to adoption of ICV in Nigeria. According to FAOSTAT (2016), the estimated area under cowpea in 2016 was 3.6 million ha and a total of 2.3 million households grew cowpea in the same year. In our sample, the estimated area under cowpea for each household was 1.58 ha while the household size was 8.3. Combining all these parameters, the 5 percentage-point reduction in poverty (Fig. 1) translates to about 929,450 farmers lifted out poverty. The results for the other poverty lines can be estimated and interpreted in a similar manner. Similarly, about 971,310 people have been lifted out of asset poverty due to the adoption of ICV.

## 5 Conclusions and policy implications

Poverty reduction is an important policy objective for many developing countries including Nigeria. Through their yield-enhancing and income-increasing effects, the adoption of improved cowpea varieties offers a considerable promise in this area. However, empirical evidence that shows the impact of ICV on poverty is rather limited in Nigeria. Using a comprehensive household and plot level data from over 1500 households, this study analyzed the impact of adoption of improved cowpea on household income, asset ownership, poverty and asset poverty.

Our endogenous switching regression results show that after accounting for both observed and unobserved heterogeneity, adoption was associated with an increase in household income and asset ownership by 17% and 24% respectively. Results from the counterfactual analysis indicate that adoption of ICV reduced poverty and asset poverty on average by 5 percentage points. This result is important in particular because it shows that adoption of improved cowpea not only increases income and asset holdings, but also reduces income poverty and asset poverty.

The finding of a positive and significant effect of information and the years the farmers have been aware of improved varieties on the decision to adopt suggests that improving access to information on improved cowpea varieties would help in enhancing their adoption and diffusion in Nigeria. This is important because the poverty-reducing effects of ICV are expected to grow with increasing adoption. In this regard, considerable investments should be made to strengthen and improve the cowpea seed systems to ensure that improved seeds are readily available at affordable prices to the smallholder farmers.

## Declaration of Competing Interest

None.

## Figures and Tables

**Table A1:** Improved cowpea varieties considered in the study.

Variety	Year variety released
IT89KD-288 (Sampea-11)	2009
IT99K-216-24-2	Not yet released
UAM09-1055-6 (Fuampea 1)	2016
IT90K-277-2 (Sampea 9)	2005
IT99K-573-1-1 (Sampea-14)	2011
IT98K-491-4	Not yet released
IT97K-499-35 (Sampea-10)	2008
IT98K-573-2-1 (Sampea 15)	2011
IT07K-292-10 (Sampea 16)	2015
IT98K-205-8	Not yet released
IT89KD-391 (Sampea 12)	2009
IT93K-452-1 (Sampea 8)	2005
IT98K-131-2	Not yet released
IAR48 (Sampea 7)	1986
UAM09-1046-6-1	Not yet released
IT07K-318-33 (Sampea 17)	2015

**Table A2:** Parameter estimates for the test on the validity of the selection instrument.

Variable	Adoption of ICV	Household income	Asset ownership
Age of the household head	−0.032* (0.017)	−0.051*** (0.012)	−0.009 (0.017)
Age of the household head squared	0.000* (0.000)	0.000*** (0.000)	0.000 (0.000)
Sex of the household head	0.286 (0.206)	0.087 (0.114)	0.227 (0.163)
Education	0.463** (0.187)	−0.078 (0.154)	−0.444** (0.221)
Number of male adults	−0.017 (0.028)	−0.112*** (0.018)	−0.077** (0.026)
Number of female adults	0.023 (0.037)	−0.115*** (0.024)	−0.075** (0.035)
Ln cultivated land	−0.028 (0.055)	0.065* (0.035)	0.295*** (0.051)
Access to off farm income	0.111 (0.100)	0.472*** (0.066)	−0.082 (0.095)
Crop marketing	0.116 (0.417)	0.044 (0.291)	0.020 (0.417)
Implement index	0.026 (0.031)	0.073*** (0.020)	0.345*** (0.029)
Information index	0.045* (0.025)	0.038** (0.019)	0.013 (0.027)
Number of donkeys	0.050 (0.075)	−0.024 (0.056)	0.104 (0.081)
Credit constrained	−0.011 (0.078)	−0.124** (0.052)	−0.176** (0.074)
Time to output market	−0.000 (0.001)	0.000 (0.000)	0.001 (0.001)
Ln distance to seed market	0.010 (0.034)	−0.015 (0.024)	0.051 (1.490)
Years aware	0.212*** (0.015)	0.007 (0.790)	0.014 (0.012)
Northeast	−0.168** (0.078)	0.103** (2.050)	0.149** (0.072)
Constant	−0.454 (0.458)	1.455*** (4.710)	5.037*** (0.444)
Observations	1525	892	892

Standard errors in parentheses.

* p < 0.10.

** p < 0.05.

*** p < 0.001.

## Footnotes

1 We use the abbreviation “FGT” indices in the subsequent section.

2 Ferreira et al. (2016) provides more details on how this poverty line was constructed.

3 The focus of this study was mainly to estimate the impacts of adoption of ICV on income, asset ownership and poverty and therefore we are not going to discuss these results.

4 This was calculated as 1.9*0.04*365.
